# Improving outpatient safety through effective electronic communication: a study protocol

**DOI:** 10.1186/1748-5908-4-62

**Published:** 2009-09-25

**Authors:** Sylvia J Hysong, Mona K Sawhney, Lindsey Wilson, Dean F Sittig, Adol Esquivel, Monica Watford, Traber Davis, Donna Espadas, Hardeep Singh

**Affiliations:** 1 Houston VA HSR&D Center of Excellence and The Center of Inquiry to Improve Outpatient Safety Through Effective Electronic Communication, Michael E DeBakey Veterans Affairs Medical Center and the Section of Health Services Research, Department of Medicine, Baylor College of Medicine, Houston, Texas, USA; 2 University of Texas School of Health Information Sciences, Houston, Texas, USA; 3 University of Texas — Memorial Hermann Center for Healthcare Quality and Safety, Houston, Texas, USA

## Abstract

**Background:**

Health information technology and electronic medical records (EMRs) are potentially powerful systems-based interventions to facilitate diagnosis and treatment because they ensure the delivery of key new findings and other health related information to the practitioner. However, effective communication involves more than just information transfer; despite a state of the art EMR system, communication breakdowns can still occur. [1-3] In this project, we will adapt a model developed by the Systems Engineering Initiative for Patient Safety (SEIPS) to understand and improve the relationship between work systems and processes of care involved with electronic communication in EMRs. We plan to study three communication activities in the Veterans Health Administration's (VA) EMR: electronic communication of abnormal imaging and laboratory test results via automated notifications (*i.e.*, alerts); electronic referral requests; and provider-to-pharmacy communication via computerized provider order entry (CPOE).

**Aim:**

Our specific aim is to propose a protocol to evaluate the systems and processes affecting outcomes of electronic communication in the computerized patient record system (related to diagnostic test results, electronic referral requests, and CPOE prescriptions) using a human factors engineering approach, and hence guide the development of interventions for work system redesign.

**Design:**

This research will consist of multiple qualitative methods of task analysis to identify potential sources of error related to diagnostic test result alerts, electronic referral requests, and CPOE; this will be followed by a series of focus groups to identify barriers, facilitators, and suggestions for improving the electronic communication system. Transcripts from all task analyses and focus groups will be analyzed using methods adapted from grounded theory and content analysis.

## Background

Many errors in health care relate to lack of availability of important patient information. The use of information technology (IT) and electronic medical records (EMR) holds promise in improving the quality of information transfer and is key to patient safety [4]. For instance, the Veterans Health Administration's (VA) EMR, also known as the Computerized Patient Record System (CPRS), uses the 'view alert' notification system, a communication system which immediately alerts clinicians about clinically significant events such as abnormal diagnostic test results. Similarly, referrals in CPRS are entered through computerized provider order entry (CPOE) and may overcome previously described communication breakdowns in the referral process [5,6]. Both these strategies could potentially reduce delays in diagnosis and/or treatment. Other types of electronic communications in the CPRS include prescription transmission, also through CPOE, which can improve communication between providers and pharmacists. Several studies have found the use of CPOE systems reduce medication errors and overall patient harm [7-10].

Health IT and EMRs are perhaps one of the most powerful systems-based interventions to facilitate the diagnostic process because they ensure the delivery of key findings and other health-related information to the practitioner [11]. However, as we have discovered, effective communication involves more than just information transfer. Despite a state of the art EMR system, such as the VA's CPRS, we have found new types of communication breakdowns [2,3]. For instance, we recently evaluated communication outcomes of abnormal diagnostic lab and imaging test result alerts and found 7% and 8%, respectively, to lack timely follow-up. We also found breakdowns among communication of electronic referrals [Singh H, Esquivel A, Sittig DF, Schiesser R, Espadas D, Petersen LA.: Follow-up of electronic referrals in a multispecialty outpatient clinic,. Manuscript submitted in 2009].

**Figure 1:**
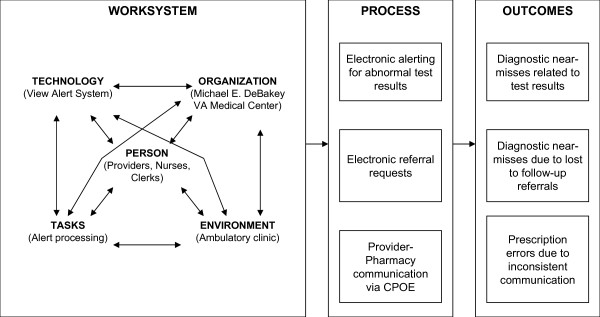
**A conceptual framework to understand and improve the view alerts system (Adapted from SEIPS)**.

To improve the design of systems, the Institute of Medicine has proposed the application of engineering concepts and methods, especially in the area of human factors [9,12]. For example, overlooking abnormal test results despite reading them, and prescriptions with errors despite CPOE, may suggest problems with how the tasks are structured, and not necessarily with the quality of medicine being practiced; thus, these examples underscore the need to look beyond clinical science for a solution to the problem [13,14]. In order to identify points for improvement and to design interventions that facilitate human-computer interaction [15], usability engineering approaches, that is, using engineering principles to make computer interfaces easier to interact with [16], are needed to assess and improve electronic communication. In this project, we will adapt a model developed by the Systems Engineering Initiative for Patient Safety (SEIPS) [17] to understand and improve the relationship between work systems and processes of care involved with electronic communication in CPRS (Figure 1). The SEIPS model integrates Donabedian's Structure-Process-Outcome framework to improve quality [18] and provides a comprehensive conceptual framework for application of systems engineering concepts to electronic communication. We believe this adaptation will lead to better design of interventions grounded in human factors aimed at improving patient safety related to electronic communication breakdowns. We plan to study three communication activities in CPRS, the VA's EMR: electronic communication of abnormal diagnostic test results such as imaging and laboratory; electronic referral requests; and provider-to-pharmacy communication via CPOE.

Breakdowns in these three processes can lead to diagnostic and medication errors, which are common types of safety concerns [19-24]. We will conduct usability testing of electronic communication systems and redesign the work system to improve care processes. Our specific aim is to evaluate the systems and processes affecting outcomes of electronic communication in CPRS with regards to communication of abnormal tests results, electronic referral requests, and provider-to-pharmacy communication via CPOE using a human factors engineering approach, and hence guide the development of interventions for work system redesign.

In this protocol, we describe methods adapted from human factors and psychology to analyze the ways in which providers currently use CPRS to communicate in each of the three discussed areas and to identify barriers to effective electronic communication.

## Methods

### Clinical setting

This study will take place at a large tertiary care, academically affiliated VA Medical Center in the Southwest. This medical center has been equipped with CPRS (as is now the case at all VA facilities) for more than ten years, and uses CPOE and electronic transmission of laboratory and diagnostic imaging tests, referrals, and medication prescriptions. Because of the electronic nature of CPRS, it is possible to track many features of all electronic requests, including the ordering provider, date of order and completion, and the date the resulting alert (for diagnostic tests/imaging and referrals) was issued and received follow-up.

### Design

This research will consist of various task analyses to identify potential sources of error related to the three electronic communication activities described earlier: diagnostic test result alerts, electronic referral requests, and provider-to-pharmacy communication via CPOE. The task analyses will be used to inform the focus groups by identifying barriers, facilitators, and suggestions for improving the electronic communication system.

The proposed two-pronged approach to study all three communication activities uses task analytic techniques initially to ascertain how each process was actually being managed. The second phase of our method employs focus groups to identify barriers, facilitators, and suggestions for improving each activity. However, due to the different nature of each communication activity, the specific task analytic techniques and focus group sampling frames will vary from activity to activity. Table 1 summarizes the data collection and analysis plans for all three-communication activities.

**Table 1:** Summary of research design by content domain

	**Electronic communication of abnormal diagnostic test results**	**Electronic referral requests**	**Provider-to-pharmacy communication via CPOE**
**Task Analysis**			

**Sample**	Primary care providers (50% timely and 50% untimely follow-up)	Specialists from five clinics	Primary care providers (50% high and 50% low prescription error)

**Procedure**	Task-based interviews on current knowledge and use of CPRS alert management features	Cognitive walkthrough of consult process at each specialty	Think aloud exercise of commonly miss-entered prescriptions

**Analysis**	Content analysis of alert management schedules, knowledge of alert management features, and use of workarounds	Process map of consult process at each specialty; corroboration against independent primary care task database	Content analysis of think aloud transcripts for correctness of prescription entry and specific strategies used

**Focus Groups**			

**Sample**	Primary care, laboratory, and IT personnel	Primary care providers, specialists, and IT personnel	Primary care providers, IT personnel, and pharmacists

**Procedure**	Three focus groups: Providers with timely follow-up (fresh data collection), Providers with untimely follow-up (fresh data collection) Mix of providers with timely and untimely follow-up (member checking and corroboration)	Four focus groups: Primary care providers (fresh data collection) Specialists (fresh data collection) Primary care providers (member checking and corroboration) Specialists (member checking and corroboration)	Three focus groups of pharmacists and: Providers with high prescription conflict errors (fresh data collection), Providers with low prescription conflict errors(fresh data collection) Mix of providers (member checking and corroboration)

**Analysis**	Grounded theory analysis of focus group transcripts; inductive coding taxonomy development via single sequence of coding, validation, and consensus; taxonomy fitted to SEIPS^a ^model and used for open, axial, and selective coding	Grounded theory analysis of focus group transcripts; inductive coding taxonomy development via iterative process of coding, validation, and consensus; taxonomy fitted to SEIPS model and used for open, axial, and selective coding	Grounded theory analysis of focus group transcripts; inductive coding taxonomy development via single sequence of coding, validation, and consensus; taxonomy fitted to SEIPS model and used for open, axial, and selective coding

^a^Systems Engineering Initiative for Patient Safety

### Sample selection

Participants will be sampled according to rates of communication breakdowns; for example, rate of lack of untimely follow-up after defined time-intervals, or frequency of CPOE transmitted prescriptions with inconsistent communication. We recently studied the rates of these communication breakdowns at a multispecialty VA ambulatory clinic by reviewing patient charts in CPRS [2,3,25]. The results from the medical record reviews will be used to classify providers into groups, which will form the sampling pool for each of the three communication activities that are the focus of the present study. For example, providers with two or more diagnostic tests results alerts without follow-up after four weeks, or with two or more prescriptions transmitted via CPOE with inconsistent communication, counted separately for each domain, will be classified as high error. Similarly, providers with one or fewer alerts lacking timely follow-up at four weeks, or with less than two prescriptions with inconsistent communication will be classified as low error. Within each group, we will sample trainees (residents and fellows), attending physicians, and allied health professionals (physician assistants and nurse practitioners). For electronic referral requests, we will sample referring providers consulting each of five high-volume specialty services: cardiology, gastroenterology, neurology, pulmonary, and surgery. Specialists will be purposively sampled according to their involvement and expertise in the referral process in their respective specialty service.

### Task analysis

Because the nature of resulting errors varies for each communication activity (*e.g.*, errors of omission result for diagnostic tests results alerts and electronic referrals requests, whereas provider-to-provider communication via CPOE errors can potentially result in the wrong medication rather than no medication being dispensed), we will use different interview procedures based on techniques used in cognitive task analysis to study all three communication activities. In all cases, interviews will be conducted by an interviewing team composed of a lead interviewer and a secondary note-taker who will capture responses and make field notes as the interview occurs. All interviews will be audio recorded with the participants' consent; interview recordings will later be transcribed for analysis. In all cases, the results of the task analysis will be used to develop the question content for the focus groups. Below we describe the procedure and data analysis plan for the task analysis of each communication activity.

#### Task analysis procedures

##### Diagnostic test results alerts

We will interview each provider independently on how they manage abnormal diagnostic test results alerts received in CPRS, we will pay particular attention to the strategies they use to manage their view alert window on a daily basis. We will also focus on existing alert management features in CPRS, including the ability to customize notification settings to reduce alerts that the provider feels are unnecessary; the ability to sort alerts for faster and easier processing; appropriate use batch processing of alerts; and the ability to alert additional providers on a particular test result when the ordering provider is not in office. Appendix 1 lists the questions asked of each participant.

##### Electronic referrals requests

We will interview each participant independently, and ask them to walk a naïve user through the process of receiving, processing, and completing a referral. (Appendix 1 lists the questions to be asked of each participant).

##### Provider-to-pharmacy communication via CPOE

We will interview each participant independently using a think aloud procedure (also known as a verbal protocol) [26]. This is a technique whereby the subject performing a task verbalizes all of the steps involved in performing the task in real time, as he/she performs the task -- this includes any mental processes and information considered during task performance; in essence 'thinking aloud' as the task is performed. This technique is particularly useful for tasks involving heavy cognitive processing, and captures many components of the task not directly observable by a task analyst. Based on the most commonly observed prescription entry errors reported by Singh *et al. *[25], five scenarios will be created to observe providers' strategies for entering these commonly error-prone prescriptions.

#### Analysis

##### Diagnostic test results alerts and provider-to-pharmacy communication via CPOE

We will use qualitative techniques adapted from grounded theory[27] and content analysis [28] to identify patterns in how participants manage their diagnostic test results alerts and how providers enter complex prescriptions in CPRS to communicate with the pharmacy. This includes the development of an initial coding taxonomy, open coding (where the text passages will be examined for recurring themes and ideas), artifact correction and validation, and quantitative tabulation of coded passages.

##### Coding taxonomy development

Immediately after each interview, the interviewing team will organize and summarize the responses from each interviewee into a structured data form to develop an initial taxonomy to be used in coding the full transcripts. An industrial/organizational psychologist experienced in task analysis and qualitative research methods will develop the initial code set; to minimize bias, the code developer will not conduct the interviews during data collection.

##### Coder training

Coders will attend an educational session where they will be instructed on the alerts and prescription entry interfaces in CPRS, the details of the coding taxonomy, and the basics of coding in Atlas.ti [29], a qualitative data analysis software package based on Strauss and Corbin's grounded theory methodology [27]. After the educational session, each coder will independently code a training transcript; the team will then reconvene to calibrate their responses.

##### Open coding

Two coders will independently code the interviews using the initial taxonomy developed from the response summaries. Coders will be required to use the existing taxonomy first, but may create additional codes should material worth capturing appear in the transcripts that does not fit into any of the existing code categories.

##### Artifact correction and validation

The two independent coding sets will be reviewed by a third coder for correcting coding artifacts, validation, and inter-rater agreement. The goal of correcting coding artifacts is to prepare the two independent coders' transcripts for validation and facilitating the calculation of inter-rater agreement. This involves: mechanically merging the two coders' coded transcripts using the Atlas.ti software (so that all data appears in a single, analyzable file); identifying and reconciling nearly identical quotations that were assigned the same codes by each coder (*e.g.*, each coder may capture a slightly longer or shorter piece of the same text); and correcting misspellings or extraneous characters in the code labels.

Through the validation process we will ensure pre-existing codes are used by both coders in the same way, reconcile newly created codes from each coder that referred to the same phenomenon but were labeled differently, and resolve remaining coding discrepancies. For quotations that do not converge (*i.e.*, do not receive identical codes from each coder), the validator will identify quotations common to both coders receiving discrepant codes, and select the best fitting code, as well as identify discrepant quotations (*e.g.*, quotations identified by one coder but not the other). Discrepant quotations will be resolved by discussion and team consensus.

##### Code tabulation and statistics

We will tabulate the number of quotations identified from each participant about each code. We will use this tabulation to calculate descriptive statistics of the alert management strategies employed by participants, as well as non-parametric statistics to identify differences in the alert management strategies of high and low error providers. Our purpose for reporting descriptive and non-parametric statistics from code tabulation is largely based on our research question to compare the strategies used by the high error and low error provider groups in how they manage their view alerts. We will conduct similar analyses for coded CPOE transcripts.

##### Electronic referral requests

The interviewing team will organize and summarize the responses from the interviewees to capture the basic course of action for processing a referral from beginning to end for each specialty, including roles assigned to specific personnel (*e.g.*, who reviews incoming referrals), task completion criteria (*e.g.*, criteria for returning the referral request to the ordering provider without completing the request), potential bottlenecks, and process points conducive to loss of follow-up. We will use these summaries to create a separate process map for each specialty. We will then compare the process maps from each specialty to identify process differences across specialties.

As an external check for the validity of the process maps, the tasks in the process maps will be cross-checked against referral tasks from a validated task database for VA primary care, generated by independent sources [30]. Details of the purpose and creation of this task database have been published elsewhere [31,32] Although this task database was developed to describe primary care tasks, rather than specialty tasks, one of the most commonly performed activities in primary care is placing and following up on referral requests. Consequently, if the specialty process maps validly and completely capture the referral process, a significant number of the referral tasks in the task database should be present in the process maps.

### Focus groups

#### Participants and sampling frame

We will conduct three to four focus groups for each of the three communication activities; each focus group will consist of six to eight participants each, the recommended size for semi-structured focus groups [33]. Primary care participants will include trainees, attending physicians, and allied health professionals.

To study electronic communication of diagnostic test results alerts, we will purposively sample and sort primary care personnel into focus groups according to their rates of timely follow-up to alerts, as was done with the task analysis sampling frame. Laboratory and IT personnel will also participate in the diagnostic test results alert focus groups. The first focus group will contain providers with high rates of timely follow-up; the second, providers with low rates of timely follow-up; the third, a mix of providers.

To study electronic referrals requests, we will conduct four focus groups. Two focus groups will consist of referring primary care providers; the other two focus groups will consist of specialists from the five specialties sampled in the task analysis.

To study provider-to-pharmacy communication via CPOE, we will conduct three focus groups. One will consist of primary care providers, a second one will consist of pharmacists, and a third one will consist of both pharmacists and providers. An IT representative will be invited to all three focus groups.

#### Focus groups procedure

Three research team members will be present at each focus group: an experienced facilitator, the primary note taker (a research team member with a background in qualitative methods), and a clinician, to provide clarification and context as needed. For the first two focus groups, we will ask participants to discuss barriers and facilitators to successfully managing and following up on alerts and referrals, and entering medications in CPRS, and to provide suggestions for improving the way to accomplish these. Our goal will be to discuss perceptions, needs, experiences, and problems but most importantly potential best strategies for improvement. We will encourage participants to think beyond the CPRS interface, and to consider the factors of the adapted SEIPS model as a guide to think broadly. The adapted SEIPS model (Figure 1) will guide the focus group according to its components (*e.g.*, organizational, environmental, technological, task-related, and personnel factors). Based on the field notes of the first two focus groups in each domain, we will present the participants of the subsequent focus groups the most frequently raised barriers, facilitators, and suggestions for improvement, checking for agreement and asking for additional detail where appropriate. Participants from the subsequent focus groups will also be encouraged to volunteer their own barriers, facilitators, and suggestions for improvement if they have not already been mentioned in the previous two groups. Initial protocols for the focus groups appear in Appendix 2. In the case of the referrals focus groups, primary care providers in the subsequent group will hear content from the specialists' focus group and vice versa, in order to cross-check the referral process from both perspectives.

#### Data analysis

We will use qualitative techniques adapted from grounded theory [27] and content analysis [28] to analyze our focus groups and identify common barriers and facilitators for each domain. Techniques will include the development of an initial coding taxonomy, open coding (where the text passages were examined for recurring themes and ideas), axial coding (where themes were related into a conceptual model), and selective coding (the identification of a core category that best summarizes the data).

##### Coding taxonomy development

Two coders will independently code transcripts from the focus groups, looking for instances of barriers, facilitators, and suggestions for improvement. The two independent coding sets will then be reviewed by a third coder with a clinical background to correct coding artifacts (see task analysis data analysis section for alerts above for more details), and identify codes needing additional processing, such as codes with unclear labels or definitions, pairs/sets of codes that are too specific and could be merged into a single code, or codes that are too general and could be split into multiple codes. The coding team will then review these candidates and based on group discussion, will re-label, split, or merge codes as necessary. The end product of this process will be a single file with a list of quotations and coding taxonomy the coding team agrees accurately represents the corpus of the focus group data.

##### Open Coding

After a one-week waiting period to reduce the effects of priming, the coders will each independently code the clean quotation list using the final coding taxonomy developed through the validation process. Coders will be required to use the existing taxonomy, and will not be permitted to add new codes. Cohen's Kappa will be used to compute inter-coder agreement, as an estimate of the extent to which the codes are crisply defined.

##### Conceptual model fit

We are interested in exploring the extent to which the issues raised during the focus groups are consistent with existing models of work systems and patient safety, specifically the adapted SEIPS model. To that end, the final code list will be categorized according to the five factors proposed in the model to check the fit of the emergent codes with model's existing taxonomy. Codes that cannot be cleanly categorized into one of the five factors will be identified as 'uncategorizable'. We will then calculate the percentage of categorizable codes, and examine the distribution of codes into the factors of the model to ascertain which factors are most influential in these data.

##### Axial coding

The coded passages from the focus groups will first be organized according to groundedness (*i.e.*, the number of quotations to which a code was assigned) to determine the most salient themes in the data. Using the constant comparative approach [27], the salient themes will then be organized to identify the causal, contextual, and intervening conditions that best explain barriers to effective alert management, referral management, and CPOE; suggestions for improvement will be linked with relevant categories as well.

##### Selective coding

Once the codes are organized and thematically related, we will seek to identify a central category that best summarizes either the central problem or the relationships observed in the data. All other substantive categories or themes will be organized around this central category.

## Discussion

Using the proposed human factors engineering approach, our studies based on these methods will provide a foundation to develop and apply multidisciplinary interventions to redesign communication processes within an EMR. Our findings will identify barriers, facilitators, and strategies for improvement in electronic communication through CPRS and inform the design of other EMR improvements in the future.

Abnormal test results are highly prevalent in the VA patients, and their timely follow-up is essential. Hence, our protocol has potential to improve the safety and timeliness of care for millions of veterans. Current literature and the recent VHA Directive 2009 to 2019 suggests that missed tests results are a significant patient safety concern in the VA population. For instance, a VA survey also found providers commonly reported clinically important treatment delays associated with missed test results [34].

Our studies, based on these methods, will be the first to analyze breakdowns in elecronic referral communication and lead to improvement in processes related to referrals. Similarly, recently described inconsistent communication in CPOE needs further study to reduce its potential for patient harm.

## Competing interests

The authors declare that they have no competing interests.

## Authors' contributions

SH is the study's qualitative core lead; she designed the methodological and analytic strategy for the task analyses and focus groups; she will facilitate the focus groups; lead the data analysis for task analyses and focus groups for alerts and CPOE, and provide workflow and task analysis expertise. MS will lead the validation for alerts and CPOE, aid in the analysis phase of all three communication activities, and provide clinical expertise. LW will code all transcripts, and aid in the interpretive phase of analysis. DS provided expertise on clinical informatics and will help analyze focus group transcripts during axial and selective coding. MW will code all pharmacy and referral transcripts, and aid in the interpretive phase of analysis. AE will lead the execution of data analysis for the referral domains, based on SH's analytic strategy, and provide informatics expertise with particular emphasis on referrals. TD will code alert and referral transcripts. DE is the study coordinator; she coordinated the chart review study that resulted in sampling classifications for this study, and will conduct the task analyses for all three domains, and coordinate the chart review. HS is leading this study; he was responsible for the overall design and supervision of this study and the medical record reviews that resulted in sampling classifications. All authors read and approved the final manuscript.

## Appendix 1: Task analysis questions

### Electronic communication of abnormal diagnostic test results -- task analysis

1. How do you manage your alerts? (What do you do daily, how many?)

2. Are you familiar with how to use 'Notification' - turning on or off non-mandatory alerts? If yes, how do you use this feature?

3. Do you know how to sort the alert list? Can you demonstrate?

4. Are you familiar with the 'process all' feature? If you use this feature, explain how.

5. Are you familiar with the alert when result feature?

6. Are you familiar with surrogates? OR Do you ever set a surrogate when you go on vacation? (Do you ever change your notifications when you assign a surrogate to decrease the volume of alerts going to your colleague?)

### Provider-pharmacy communication via CPOE -- think-alouds

For this study, we would like you to enter five specific prescriptions, and walk us through the process in real time as you are entering them in CPRS. As you're entering each prescription, please be specific about narrating out loud what you are selecting on screen and why. We will try to be as unobtrusive as possible, however, we may ask you to elaborate or give more detail about what you are doing if we have questions or something is unclear.

### Electronic referral requests -- cognitive walkthrough (cardiology, GI, pulmonary, neurology)

1. What is the first action when a consult is received?

2. What are the prerequisites for accepting a consult?

3. Who are the key players in processing consults for the section?

4. Walk through processes:

a. Pending

b. Accepting

c. Initial processing

d. Scheduling

e. Discontinuing

f. Completing

g. Closing out

5. What actually happens vs. what is supposed to happen?

## Appendix 2: focus group protocol

### Electronic communication of abnormal diagnostic test results alerts

1. What are some of the factors or things that you think are hindrance to effectively and efficiently processing your alerts? (Probes: Not receiving all alerts as PCP, routing alerts to the correct provider, disappearing alerts after 15 days).

2. What factors or things do you perceive as being helpful or facilitating to effectively and efficiently processing your alerts? (Probes: Using sorting features, customizing your interface, piece of paper, etc.).

3. What kind of changes would you suggest to improve the process of managing your electronic alerts? (Probes: features to track specific patients, training, separate windows to separate critical alerts).

### Electronic referral requests

#### Questions for providers (first focus group with PCP's)

1. In general, how do you know when a referral has been completed?

a. What systems if any do you have in place to follow-up on unresolved referrals (or do you just rely on the alerts)

b. What do you do once you find out that a referral you placed is unresolved?

2. Can anyone provide an example of a referral that was placed, unresolved, that resulted in harm to the patient?

a. What was the situation?

b. What do you think prevented it from getting it resolved?

c. What did you do once you found out?

d. What was the eventual outcome?

3. What are some of the barriers to getting these referrals resolved?

4. When you place a referral, how do you decide what kind of information to include in the referral request?

5. Do you receive alert notifications for discontinued referrals?

a. How often do you receive alerts for referrals that were discontinued inappropriately?

b. What do you do if a referral was inappropriately discontinued?

6. Can anyone provide an example of a referral that was discontinued, or that resulted in harm to the patient?

a. What was the situation?

b. What do you think happened in this instance?

c. What did you do once you found out?

d. What was the eventual outcome?

7. Can anyone provide an example of a referral that was completed, but not to your satisfaction?

a. What was the situation?

b. What was unsatisfactory about how the referral was completed?

c. What did you do once you found out?

d. What was the eventual outcome?

8. How do you manage referrals that were completed without scheduling a patient visit?

9. What kinds of changes would you suggest to improve the referral process?

#### Questions for providers (second focus group with PCP's)

1. Would you want to track your referrals on a monthly basis?

2. How in-depth would you prefer if referral tracking (*i.e.*, pending, cancelled, discontinued, completed) was made available?

3. Would you like to have feedback regarding referrals?

a. Individual feedback from specialists on what changes can be made to improve the process?

b. Volume feedback on how many referrals each provider placed?

4. Do you receive alert notifications for discontinued referrals?

a. How do you manage referrals that were discontinued inappropriately?

b. What do you do if a referral was inappropriately discontinued?

5. Should discontinued referrals be made a mandatory alert?

6. Do you think consultants should be incentivized?

a. (If so), what form should that incentive take?

b. (If not) Why not? What would be a better solution?

7. What level of specificity should go into a referral request? For example, if you were teaching a medical student to write up a referral, what would you tell him/her?

8. Do you feel that having a guideline for each referring service would be a helpful tool to use in your practice? (*e.g.*, a list of the top ten things to know about frequently consulted services)

9. Are you familiar with the policy on patient no-shows? What, to your understanding, is the policy on no-shows?

10. How many no-shows before the referral is discontinued?

11. After a patient does not show to an appointment, who is responsible to follow-up with that patient?

12. We have heard suggestions from providers in how to improve the referral process. This is your opportunity to add any suggestions that we may not have already mentioned. We are looking specifically for kinds of things we can change that will improve the way referrals are managed in the VA.

#### Questions for specialists (first and second focus group with specialists)

1. No shows: What is the policy? How do you handle patient no shows?

2. Calling patients: Do you usually call the patient?

3. Unresolved referrals: How do you manage these?

4. Completed referrals: How do you track the wait time?

5. Alerts: Are you aware that primary care providers do not receive an alert for discontinued referrals? Do you have any suggestions regarding alerts?

6. Improving communication: Explain what communication you have with providers and what can be done to improve communication.
